# The Effects of High-Intensity Interval Exercise on Skeletal Muscle and Cerebral Oxygenation during Cycling and Isokinetic Concentric and Eccentric Exercise

**DOI:** 10.3390/jfmk6030062

**Published:** 2021-07-16

**Authors:** Panagiotis A. Perentis, Evgenia D. Cherouveim, Vassiliki J. Malliou, Nikos V. Margaritelis, Panagiotis N. Chatzinikolaou, Panayiotis Koulouvaris, Charilaos Tsolakis, Michalis G. Nikolaidis, Nickos D. Geladas, Vassilis Paschalis

**Affiliations:** 1 School of Physical Education and Sport Science, National and Kapodistrian University of Athens, 17237 Athens, Greece; panagiotis.pere@gmail.com (P.A.P.); echerouv@phed.uoa.gr (E.D.C.); bmalliou@phed.uoa.gr (V.J.M.); tsolakis@phed.uoa.gr (C.T.); ngeladas@phed.uoa.gr (N.D.G.); 2 Sports Excellence, 1st Orthopedics Department, School of Health Sciences, National and Kapodistrian University of Athens, 17237 Athens, Greece; info@drkoulouvaris.gr; 3 Department of Physical Education and Sport Science at Serres, Aristotle University of Thessaloniki, 62110 Serres, Greece; nvmargar@auth.gr (N.V.M.); tso.p@hotmail.com (P.N.C.); nikolaidis@auth.gr (M.G.N.); 4 Dialysis Unit, 424 General Military Hospital of Thessaloniki, 56429 Thessaloníki, Greece

**Keywords:** concentric exercise, deoxygenated hemoglobin, eccentric exercise, fatigue index, oxygenated hemoglobin, tissue saturation index

## Abstract

The aim of the present study was to study the effects of cycling and pure concentric and pure eccentric high-intensity interval exercise (HIIE) on skeletal muscle (i.e., vastus lateralis) and cerebral oxygenation. Twelve healthy males (n = 12, age 26 ± 1 yr, body mass 78 ± 2 kg, height 176 ± 2 cm, body fat 17 ± 1% of body mass) performed, in a random order, cycling exercise and isokinetic concentric and eccentric exercise. The isokinetic exercises were performed on each randomly selected leg. The muscle and the cerebral oxygenation were assessed by measuring oxyhemoglobin, deoxyhemoglobin, total hemoglobin, and tissue saturation index. During the cycling exercise, participants performed seven sets of seven seconds maximal intensity using a load equal to 7.5% of their body mass while, during isokinetic concentric and eccentric exercise, they were performed seven sets of five maximal muscle contractions. In all conditions, a 15 s rest was adopted between sets. The cycling HIIE caused greater fatigue (i.e., greater decline in fatigue index) compared to pure concentric and pure eccentric isokinetic exercise. Muscle oxygenation was significantly reduced during HIIE in the three exercise modes, with no difference between them. Cerebral oxygenation was affected only marginally during cycling exercise, while no difference was observed between conditions. It is concluded that a greater volume of either concentric or eccentric isokinetic maximal intensity exercise is needed to cause exhaustion which, in turn, may cause greater alterations in skeletal muscle and cerebral oxygenation.

## 1. Introduction

High-intensity interval training is an exercise modality that consists of repeated short periods of high, maximal, or/and supramaximal intensities, separated by intervals of either low-intensity exercise or rest [1,2]. During the last two decades, high-intensity interval training has been extensively investigated and has been proposed to be a time-efficient strategy to improve exercise performance in athletes compared to traditional endurance training [3,4,5,6]. Moreover, high-intensity interval training has been used both in healthy nonathlete individuals to improve their cardiorespiratory fitness and health status [7,8,9,10,11,12] and in patients suffering from chronic diseases [13,14,15,16].

High-intensity interval training can be performed using a wide range of exercise activities such as cycling, running, rowing, swimming, and resistance training [17]. In these activities, muscles work in a continuous shortening and a lengthening cycle, i.e., concentric and eccentric muscle contraction, respectively [18]. Recently, high-intensity interval training was also performed during pure concentric (muscle shortening) and pure eccentric (muscle stretching) muscle contractions using isokinetic dynamometers [19,20,21]. It is known that different modes of muscle contraction exhibit diverse molecular, metabolic, mechanical, and neural characteristics [22,23]. Specifically, eccentric exercise is characterized by lower energy expenditure, oxygen uptake, cardiovascular demand, motor unit recruitment, and lactate production, while exhibiting increased neuromuscular efficiency and muscle power output compared to concentric exercise performed at the same absolute work output [23,24,25,26,27,28,29,30,31,32]. On the other hand, exercise activities that consist of continuous muscle shortening and lengthening cycles cause greater mechanical work and steady-state force production compared to exercises that involve pure eccentric contractions [33,34]. The aforementioned differences between the different modes of muscle contraction are mainly attributed to the different contribution of the passive skeletal muscle elements (i.e., tendons, extracellular matrix, and serial elastic components) and the filamentous protein titin [35,36].

Contemporary near-infrared spectroscopy (NIRS) may offer accurate data acquisition during exercise regarding skeletal muscle and cerebral oxygenation. Near-infrared spectroscopy is a non-invasive method for continuous monitoring tissue oxygen availability and utilization via changes in concentration of oxyhemoglobin (O_2_Hb, O_2_ delivery), deoxyhemoglobin (HHb, O_2_ extraction), total hemoglobin (THb, local blood volume), and tissue saturation index (TSI, dynamic balance between O_2_ delivery and utilization) [37,38]. High-intensity interval cycling and running exercise causes decreased skeletal muscle oxygenation, as indicated by the greater concentration of HHb, with a concomitant lower concentration of O_2_Hb or/and a lower tissue saturation index [39,40,41,42,43,44]. Regarding different types of muscle contraction, greater alterations in muscle oxygenation were found during pure concentric compared to pure eccentric maximal exercise [45,46,47]. Cerebral oxygenation was found to be significantly decreased during exhaustive incremental cycling [48,49] and sprint interval cycling [50,51,52].

To the best of our knowledge, no study has compared both skeletal muscle and cerebral oxygenation during high-intensity interval exercise (HIIE) performed with different muscle contraction modes. Therefore, the aim of the present study was to investigate the effects of HIIE on skeletal muscle and cerebral oxygenation during cycling (active knee and hip extension when pushing the pedal and active hip flexion when pulling the pedals) and isokinetic concentric (shortening phase of muscle contraction) and eccentric (lengthening phase of muscle contraction) exercise.

## 2. Materials and Methods

### 2.1. Participants 

Twelve healthy males volunteered to participate in the study (mean ± SEM; age 26 ± 1 yr, body mass 78.4 ± 2 kg, height 176 ± 2 cm, body fat 17 ± 1% of body mass). All subjects were participating in recreational physical activities; they were not smokers and they were not suffering from any musculoskeletal injury. Participants were asked to recall whether they had performed scheduled resistance or aerobic training or unaccustomed and/or heavy exercise in the three months before the study entry (e.g., soccer game, competitive running, high-impact aerobics). Individuals who reported such activities were precluded from the study. Participants were instructed to abstain from any strenuous exercise at least two days before their participation in the study and to refrain from any heavy meal or caffeine-containing beverages for at least 2 h. Written consent was obtained from all participants after they were informed of the procedures and risks of the study. The experimental procedures were in accordance with the Helsinki declaration of 1975, as revised in 2000. The intervention protocol used in the present investigation was evaluated and approved by the ethics committee of the School of Physical Education and Sport Science of the National and Kapodistrian University of Athens (Protocol #1099; 13 February 2019).

### 2.2. Experimental Procedures

The participants visited the laboratory on four separate occasions. During the first visit, they performed both the anthropometric measurements and a familiarization exercise session of both cycling and isokinetic concentric and eccentric muscle contractions. During the anthropometric measurements, the subjects were barefoot and lightly dressed, while their body mass was measured to the nearest 0.1 kg and body composition was analyzed by Bioelectrical Impedance Analysis technology (Tanita MC-780MA S, Tanita Corporation of American Inc., Arlington Heights, IL, USA). Standing height was measured to the nearest 1 cm (SECA 206, Seca Corporation, Birmingham, UK). The familiarization cycling session consisted of a 15 min warm-up, followed by three high-intensity sprints of approximately 4 s using a cycle ergometer (894E speed bike, Monark, Stockholm, Sweden). The familiarization isokinetic session was performed on an isokinetic dynamometer (Biodex, System 4 Pro, Shirley, NY, USA) and consisted of five repetitions of very low intensity followed by 3 high-intensity repetitions of both eccentric and concentric muscle contractions.

During the following three laboratory visits, the participants performed in random order one of the three HIIE modes, i.e., cycling, isokinetic concentric and isokinetic exercise. The isokinetic concentric and eccentric exercise was performed on each randomly selected leg. The duration of the exercise protocols (i.e., cycling, isokinetic concentric and isokinetic eccentric) was almost equal among them. The exercise trials were performed at the same time of day and separated by at least 3 days. Prior to each exercise session, participants performed an 8 min cycling warm-up (894E speed bike, Monark, Stockholm, Sweden). To evaluate the level of fatigue during each HIIE, the fatigue index was calculated by taking the percentage difference between maximal (1st set) and minimal (last set) of the three HIIE types according to the equation [(first set—last set/first set) × 100] [53,54].

### 2.3. High-Intensity Interval Cycling Exercise Protocol

The HIIE during cycling exercise was performed using a cycle ergometer (894E speed bike, Monark, Stockholm, Sweden) and consisted of 7 sets of 7 s maximal intensity efforts with 15 s interval between sets. Specifically, during every set, the participants were pedaling with no brake on the flywheel as fast as they could and, when the pedaling rate reached 100 rpm, a load equal to 7.5% of their body mass was dropped instantly on the flywheel (resistance equal to Wingate test). The stop-clock was starting automatically at the 100 rpm and the participants had to maintain maximal pedaling rate for 7 s while they remained seated throughout the trial. Participants were verbally encouraged to maintain their maximal effort during each set.

### 2.4. High-Intensity Interval Isokinetic Exercise Protocol

The HIIE during the isokinetic concentric and eccentric exercises was performed using an isokinetic dynamometer (Biodex, System 4 Pro, Shirley, NY, USA), and consisted of 7 sets of 5 repetitions with 15 s interval between sets, while the angular velocity was set at 60°/s. The isokinetic dynamometer was calibrated before each exercise session according to the manufacturer’s instructions. Subjects were seated (120° hip angle) with the lateral femoral condyle aligned with the axis of rotation of the dynamometer, and were coupled to the dynamometer by an ankle cuff attached proximally to the lateral malleolus. Each subject’s functional range of motion was set electronically between 20° and 100° of knee flexion (full extension 0°). Each isokinetic concentric or eccentric repetition lasted 1.34 s (i.e., knee joint range of motion 80° at an angular velocity 60°/s) or 6.7 s/set, which was almost equal to the duration of each set during cycling exercise (i.e., 7 s/set). Gravitational corrections were made to account for the effect of limb weight on torque measurements. Participants were verbally encouraged to maximally activate their knee extensors throughout each exercise bout.

### 2.5. Skeletal Muscle and Cerebral Oxygenation

Skeletal muscle and cerebral oxygenation were assessed simultaneously and non-invasively using the continuous wave NIRS technique, with two wavelengths of near-infrared light (760 and 850 nm) (Artinis Medical System, PortaMon/PortaLite, Zetten, The Netherlands). The NIRS units (i.e., PortaMon and PortaLite) consisted of multi-distance optical probes, configured with one optical receiver and three optical source emitters in order to monitor three separate regions of the tissue simultaneously. The three source emitters were on the same line as the detector. The inter-optode spacings between emitters and receiver were 30, 35, and 40 mm, and the penetration depth was approximately one-half of the distance between the emitter and the receiver (i.e., 15, 17.5, and 20 mm) [38]. The NIRS data were collected at a frequency of 10 Hz and the average values of the 3 probes were used for the data analyses. Although NIRS devices cannot discriminate between chromophores (hemoglobin and myoglobin) within the muscle, given that myoglobin content tends to remain constant during exercise, the changes in NIRS signals can be attributed to changes in hemoglobin [37,55]. The NIRS system provided muscle and cerebral changes in microvascular concentrations of oxygenated hemoglobin (Δ[O_2_Hb]) and deoxyhemoglobin (Δ[HHb]), which reflect the dynamic balance between muscle oxygen delivery and extraction in the underlying tissue [37,38]. Moreover, muscle and cerebral tissue saturation index (TSI = O_2_Hb/THb × 100) was calculated by the NIRS system using previously determined absorption and scattering coefficients [56]. Changes in total hemoglobin concentration (Δ[THb]) were calculated as the sum of O_2_Hb and HHb, while changes in [THb] were related to microvascular blood volume changes [57]. The changes (Δ) in [O_2_Hb], [HHb], [THb], and TSI were assessed from the resting values recorded over the last 30 s of a 5 min resting period preceding the start of the test. The measurements were, therefore, normalized based on these recordings (arbitrarily defined as 0 μM) and the data are presented in Δ values.

Regarding muscle oxygenation, the NIRS unit (PortaMon) was placed at the lower third of vastus lateralis muscle (≈12 cm above the patella and 5 cm lateral to the midline), after the site had been shaved and cleaned using an alcohol swab. The skinfold thickness over the vastus lateralis of both legs at the site of PortaMon placement was measured using a skinfold caliper (Harpenden, John Bull, St. Albans, UK) to determine adipose tissue thickness (i.e., skinfold thickness/2), since it may influence the amplitude of the NIRS signal [58]. In the context of the present study, NIRS measurements were not influenced by adipose tissue, since the average (±SEM) values of adipose tissue thickness for the left and the right vastus lateralis muscles were 4.49 ± 0.54 and 4.68 ± 0.50 mm, respectively, which are well below the minimum NIRS light penetration depth (i.e., 15 mm). Based on the fact that tissue oxygenation and blood flow responses may vary between different muscles and different regions of the same muscle, the probes were always positioned by the same experimenter who ensured, as much as possible, the reproducibility of NIRS placement between the two legs. Regarding cerebral oxygenation, the NIRS unit (PortaLite) was attached to the surface of the left prefrontal cortex. Participants were instructed to keep their head as still as possible during exercise to minimize the motion artifacts of the cerebral NIRS signal. Muscle and cerebral NIRS units and probes were covered with a black bandage, stabilized with tape on the cleaned skin to minimize the intrusion of external light and to prevent their movement. No sliding of the NIRS systems (i.e., PortaMon and PortaLite) was observed at the end of the three exercise sessions in all participants.

### 2.6. Rate of Perceived Exertion

Rate of perceived exertion (RPE) was assessed during high-intensity interval exercise protocols at baseline and after the 4th and the 7th set [59].

### 2.7. Statistical Analysis

The normality of all dependent variables was examined by the Shapiro–Wilk test and no significant violence of normality distribution was found (*p* > 0.05). A two-way ANOVA test was used to compare the three exercise modes in regard to performance, as well as in muscle and cerebral oxygenation [group (3 exercise modes) × time (7 sets) or (3 sets) in the case of rate of perceived exertion]. When a significant interaction was obtained, pairwise comparisons were performed through the Sidak test. When sphericity was violated, the Greenhouse–Geisser correction was applied. Data are presented as mean ± standard error of the mean (SEM) and the level of significance was set at α = 0.05. The SPSS version 21.0 was used for all analyses (SPSS Inc., Chicago, IL, USA).

## 3. Results

### 3.1. Exercise Performance

**Figure 1:**
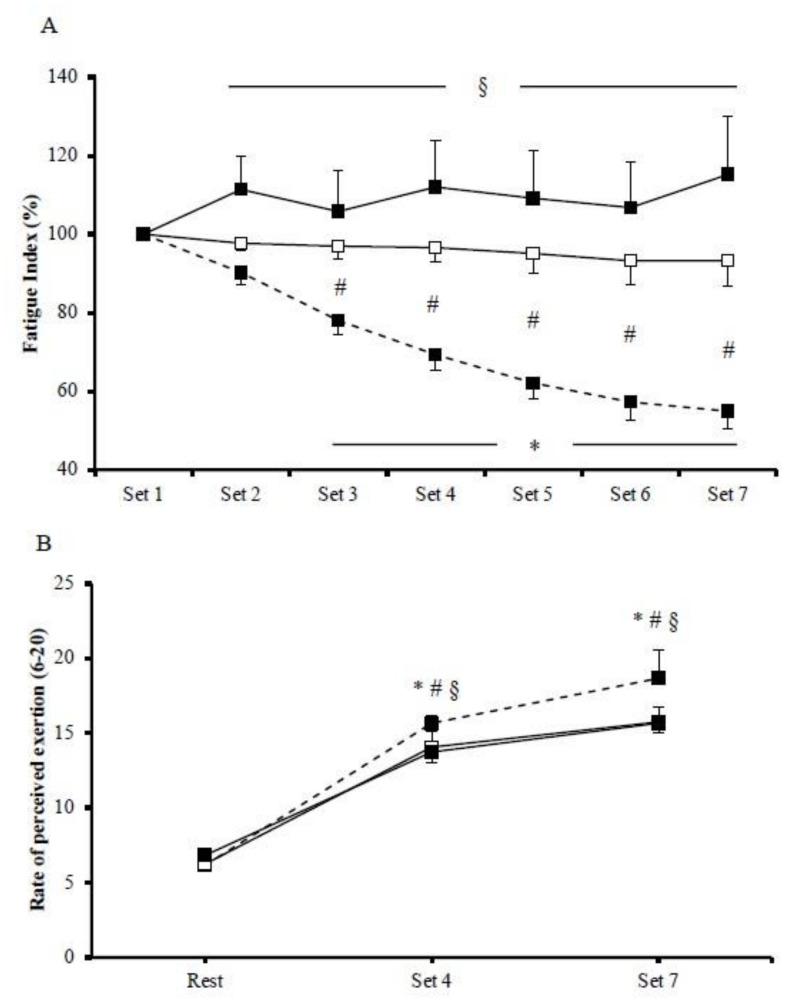
Fatigue index (**A**) and rate of perceived exertion (**B**) during the cycling (dash line), the concentric (□), and the eccentric (■) exercise. (*) indicates significant difference compared to baseline in the cycling exercise; (§) indicates significant difference between the cycling exercise and the eccentric exercise; (#) indicates significant difference between the cycling exercise and the concentric exercise.

Mean power output was significantly different among HIIE protocols. In particular, mean power output was significantly greater during cycling (585.4 ± 20.5 W; *p* < 0.001) compared to isokinetic concentric (118.1 ± 2.3 W) and eccentric (124.7 ± 4.2 W) exercise protocols. Cycling performance was gradually decreased, as expressed by fatigue index (*p* = 0.002; Figure 1A). There was a significant exercise by time interaction regarding fatigue index (*p* < 0.001). Specifically, the magnitude of the fatigue index reduction was greater in cycling compared to concentric exercise between the third and the seventh bout while, compared to eccentric exercise, the difference was greater between the second and the seventh bout. The magnitude of fatigue index between the two isokinetic modes was greater in concentric compared to eccentric exercise. Regarding RPE, there was a significant main effect of exercise protocol (*p* < 0.001), with the mean values being higher in cycling (13.5 ± 1.0) compared to concentric (12.0 ± 0.7) and eccentric (12.1 ± 0.7) exercise, whereas no difference was observed between concentric and eccentric protocols (Figure 1B).

### 3.2. Muscle Oxygenation

**Figure 2:**
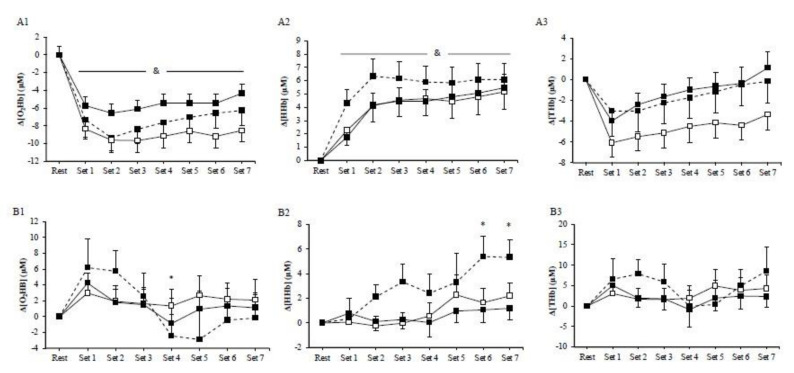
Muscle (**A**) and cerebral (**B**) oxyhemoglobin (1), deoxyhemoglobin (2), and total hemoglobin (3) during the cycling (dash line), the concentric (□), and the eccentric (■) exercise. (*) indicates significant difference compared to baseline in the cycling exercise; (&) indicates significant difference compared to baseline for all exercise modes.

**Figure 3:**
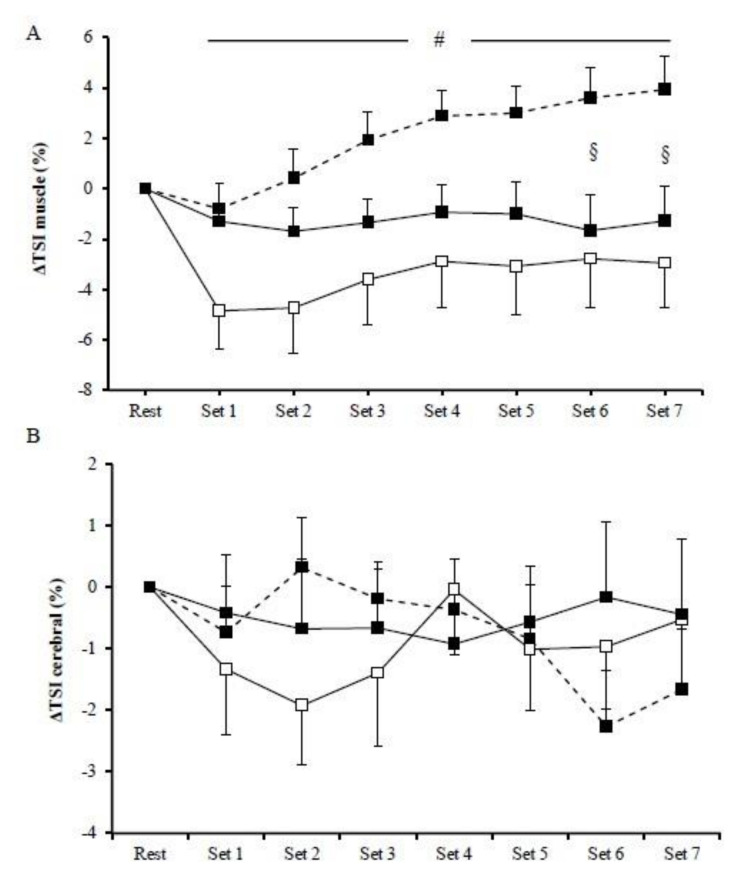
Muscle (**A**) and cerebral (**B**) tissue oxygenation index during the cycling (dash line), the concentric (□), and the eccentric (■) exercise. (#) indicates significant difference between the cycling exercise and the concentric exercise; (§) indicates significant difference between the cycling exercise and the eccentric exercise.

No significant main effects of exercise mode (*p* > 0.05) or exercise mode by time interaction (*p* > 0.05) were observed for muscle Δ[O_2_Hb], Δ[HHb], and Δ[THb] among HIIE protocols. However, the main effect of time was significant (*p* < 0.01) in all HIIE regarding muscles Δ[O_2_Hb], Δ[HHb], and Δ[THb]. In particular, muscle Δ[O_2_Hb] rapidly decreased at the initiation of exercise and remained low until the end of the exercise (Figure 2A1), whereas muscle Δ[HHb] progressively increased compared to baseline until the end of the three exercise modes (Figure 2A2). Furthermore, muscle Δ[THb] rapidly decreased at the initiation of exercise and then progressively increased and peaked at the end of the exercise protocol (Figure 2A3). There was a significant main effect of exercise (*p* = 0.01) on muscle ΔTSI, with the mean values being higher in cycling (2.14 ± 0.45%) than in concentric exercise (−3.56 ± 0.66%; Figure 3A). Muscle ΔTSI showed no statistical difference between the two isokinetic exercise modes (*p* > 0.05). Muscle TSI significant increased only during the cycling exercise (*p* = 0.007; Figure 3A).

### 3.3. Cerebral Oxygenation

No significant exercise by time interaction was observed among HIIE modes in cerebral oxygenation, as measured by Δ[O_2_Hb], Δ[HHb], and Δ[THb] (Figure 2B). However, during cycling, HIIE, cerebral Δ[O_2_Hb], and Δ[HHb] were significantly changed (*p* < 0.05). In particular, cerebral Δ[O_2_Hb] rapidly increased at the initiation of exercise, followed by a decrease at the fourth set (Figure 2B1), while the cerebral Δ[HHb] during cycling continuously increased until the end of the exercise intervention (Figure 2B2). No significant main effect of exercise was observed in the prefrontal cortex TSI (*p* > 0.05) (Figure 3B).

## 4. Discussion

In the present study, we investigated whether skeletal muscle (i.e., vastus lateralis) and cerebral oxygenation would be affected differently during HIIE of different muscle contraction modalities, such as cycling (concentric and eccentric muscle contraction during the power and the recovery phase, respectively), pure concentric, and pure eccentric exercise. It was found that the mean power output was significantly greater during cycling compared to both isokinetic concentric and eccentric exercise protocols, which could be related to the greater level of fatigue that was found during the cycling HIIE compared to concentric and eccentric exercises. Regarding muscle oxygenation, in the three modes of exercise, the Δ[O_2_Hb] and the Δ[HHb] were significantly reduced and increased, respectively, while no difference was observed between exercise modes. On the contrary, the tissue saturation index was significantly greater during cycling compared to concentric and eccentric exercises. Cerebral oxygenation was not different among the three exercise modes, based on the measured parameters (i.e., Δ[O_2_Hb], Δ[HHb], Δ[THb], and TSI).

It is known that performance can be significantly reduced during cycling HIIE; however, performance reduction has considerable variability, since it was found to range between 11% and 35% [40,41,42,60,61]. It was hypothesized that the large variability between investigations could be attributed to methodological differences including, among others, exercise duration, number of repetitions, recovery time, and physical fitness of the participants. In the present investigation, the different types of muscle action that were used led to differences in performance during the three modes of HIIE. Specifically, the magnitude of the performance reduction was greater in cycling compared to both eccentric and concentric exercises, while the performance reduction was greater in concentric compared to eccentric exercise, which is in line with studies of the same nature [45,62,63].

It is known that there is a significant decrease in muscle Δ[O_2_Hb] and an increase in muscle Δ[HHb] in response to acute high-intensity interval exercise when performed in a wide range of exercise durations (6–30 s), recovery periods (12 s–2 min), and exercise types (cycling and running) [39,40,41,42,43,44]. This is also the case in the present investigation, where the three different modes of muscle contraction during HIIE caused significant decreases in Δ[O_2_Hb] and significant increases in Δ[HHb], whereas this response (i.e., decreased Δ[O_2_Hb] and increased Δ[HHb]) is an indication of decreased muscle oxygenation [49]. However, the TSI was significant increased only in the cycling exercise compared to eccentric and concentric exercise; this is a finding which is in agreement with the greater fatigue index observed in cycling compared to eccentric and concentric exercise. In contrast, previous investigations demonstrated a significant decrease in TSI during high-intensity exercise; however, this discrepancy could be attributed to the different exercise durations (i.e., 7 vs. 30 s of maximal effort) [44,60].

The HIIE adopted in the present study did not cause any alteration in cerebral oxygenation (i.e., Δ[O_2_Hb], Δ[HHb], Δ[THb], and TSI) during the eccentric and concentric muscle actions. However, regarding cycling exercise, there was a tendency for increased Δ[O_2_Hb] during the first two sets of HIIE, which could be attributed to increased nervous activation [64]; however, during the last four sets of HIIE, the Δ[O_2_Hb] was marginally decreased. Moreover, during the cycling exercise, the cerebral Δ[HHb] gradually increased, reaching significance at the last two sets of HIIE. No significant difference was observed among the three modes of muscle action. It is clear that neither the different performance magnitude between conditions nor the different type of muscle action were adequate to affect cerebral oxygenation. The present results are partially in line with previous investigations where HIIE of either running or cycling caused decreases in cerebral [O_2_Hb] and tissue saturation index while also causing increases in cerebral [HHb] [40,43,49,50,65,66]. It seems that cerebral oxygenation is mainly affected by the level of fatigue that a HIIE bout can cause; this was not the case in the present investigation, where the eccentric and the concentric exercises did not cause significant reductions in performance.

It is known that HIIT can be performed using a wide range of exercise activities such as cycling, running, rowing, and swimming which, in turn, may cause numerous physiological adaptations in health and disease [3,4,5,6,7,8,9,10,11,12,13,14,15,16,67]. High-intensity interval training has also been used during pure eccentric and pure concentric muscle actions using an isokinetic dynamometer, causing beneficial effects in health and performance parameters [19,20,21]. In the present investigation, we compared a traditional type of HIIE (i.e., cycling) with HIIE performed during isokinetic eccentric and concentric exercise in relation to skeletal muscle and cerebral oxygenation. However, based on our findings, it seems that the volume of the adopted isokinetic protocol of either concentric or eccentric exercise (i.e., seven sets of five maximal either eccentric or concentric muscle actions with 15 s recovery between sets) was not adequate to lead to exhaustion compared to a cycling exercise of concomitant exercise duration and rest. It could be suggested that equalizing task physiological demands could affect the physiological assessments differently which, in turn, could lead to different muscle and cerebral oxygenation responses.

Based on the current results, the cycling protocol was of sufficient volume and intensity to induce performance decline, as well as alterations in muscle and cerebral oxygenation. On the contrary, the isokinetic exercise (i.e., both concentric and eccentric) did not cause an alteration in the fatigue index, probably due to the low exercise volume. Of interest is the fact that the isokinetic exercise affected muscle and cerebral oxygenation, which could be attributed to the higher intramuscular pressure induced by the isokinetic exercise; this could, in turn, lead to more potent microvascular compression [68]. Therefore, it could be suggested that a low fatigue isokinetic/resistance exercise could serve as an alternative way of training for older individuals or people who exercise recreationally in order to preserve muscle function [21].

## 5. Conclusions

The aim of the present study was to collect muscle and cerebral oxygenation data during exercise and to uncover the interaction between different physiological systems in response to different types of muscle contraction. To the best of our knowledge, this is the first study that compares skeletal muscle and cerebral oxygenation during HIIE for different modes of muscle contraction, i.e., cycling (concentric and eccentric muscle contraction during the power and the recovery phase, respectively), pure concentric, and pure eccentric exercise. The main findings of the present investigation are: (i) HIIE caused different responses in the fatigue index between the cycling exercise and pure concentric and pure eccentric isokinetic exercises; (ii) muscle oxygenation, as measured via Δ[O_2_Hb] and Δ[HHb], was significantly reduced during high-intensity interval exercise in the three conditions; however, no difference was observed between them; (iii) the tissue saturation index was greater during cycling compared to concentric and eccentric exercises; (iv) cerebral oxygenation (i.e., Δ[O_2_Hb], Δ[HHb], Δ[THb], and TSI) was only marginally affected during the cycling exercise, while no difference was observed between conditions. It is clear that a greater volume of isokinetic concentric or eccentric maximal intensity is needed to cause exhaustion which, in turn, may cause greater alterations in skeletal muscle and cerebral oxygenation.

## Data Availability

Not applicable.

## References

[1] Billat L.V. (2001). Interval training for performance: A scientific and empirical practice. Special recommendations for middle- and long-distance running. Part II: Anaerobic interval training. Sports Med..

[2] Billat L.V. (2001). Interval training for performance: A scientific and empirical practice. Special recommendations for middle- and long-distance running. Part I: Aerobic interval training. Sports Med..

[3] Engel F.A., Ackermann A., Chtourou H., Sperlich B. (2018). High-Intensity Interval Training Performed by Young Athletes: A Systematic Review and Meta-Analysis. Front Physiol..

[4] Gibala M.J., Jones A.M. (2013). Physiological and performance adaptations to high-intensity interval training. Nestle Nutr Inst Workshop Ser.

[5] Gist N.H., Fedewa M.V., Dishman R.K., Cureton K.J. (2014). Sprint interval training effects on aerobic capacity: A systematic review and meta-analysis. Sports Med..

[6] Ni Cheilleachair N.J., Harrison A.J., Warrington G.D. (2017). HIIT enhances endurance performance and aerobic characteristics more than high-volume training in trained rowers. J. Sports Sci..

[7] Batacan R.B., Duncan M.J., Dalbo V.J., Tucker P.S., Fenning A.S. (2017). Effects of high-intensity interval training on cardiometabolic health: A systematic review and meta-analysis of intervention studies. Br. J. Sports Med..

[8] Daussin F.N., Zoll J., Dufour S.P., Ponsot E., Lonsdorfer-Wolf E., Doutreleau S., Mettauer B., Piquard F., Geny B., Richard R. (2008). Effect of interval versus continuous training on cardiorespiratory and mitochondrial functions: Relationship to aerobic performance improvements in sedentary subjects. Am. J. Physiol. Regul. Integr. Comp. Physiol..

[9] Gibala M.J., Little J.P., van Essen M., Wilkin G.P., Burgomaster K.A., Safdar A., Raha S., Tarnopolsky M.A. (2006). Short-term sprint interval versus traditional endurance training: Similar initial adaptations in human skeletal muscle and exercise performance. J. Physiol..

[10] Gibala M.J., McGee S.L. (2008). Metabolic adaptations to short-term high-intensity interval training: A little pain for a lot of gain?. Exerc. Sport Sci. Rev..

[11] Gillen J.B., Gibala M.J. (2014). Is high-intensity interval training a time-efficient exercise strategy to improve health and fitness?. Appl. Physiol. Nutr. Metab..

[12] Nybo L., Sundstrup E., Jakobsen M.D., Mohr M., Hornstrup T., Simonsen L., Bulow J., Randers M.B., Nielsen J.J., Aagaard P. (2010). High-intensity training versus traditional exercise interventions for promoting health. Med. Sci. Sports Exerc..

[13] Adolfo J.R., Dhein W., Sbruzzi G. (2019). Intensity of physical exercise and its effect on functional capacity in COPD: Systematic review and meta-analysis. J. Bras. Pneumol..

[14] Giallauria F., Smart N.A., Cittadini A., Vigorito C. (2016). Exercise training modalities in chronic heart failure: Does high intensity aerobic interval training make the difference?. Monaldi Arch. Chest. Dis..

[15] Iellamo F., Caminiti G., Sposato B., Vitale C., Massaro M., Rosano G., Volterrani M. (2014). Effect of High-Intensity interval training versus moderate continuous training on 24-h blood pressure profile and insulin resistance in patients with chronic heart failure. Intern. Emerg. Med..

[16] Spee R.F., Niemeijer V.M., Wijn P.F., Doevendans P.A., Kemps H.M. (2016). Effects of high-intensity interval training on central haemodynamics and skeletal muscle oxygenation during exercise in patients with chronic heart failure. Eur. J. Prev. Cardiol..

[17] Viana R.B., de Lira C.A.B., Naves J.P.A., Coswig V.S., Del Vecchio F.B., Ramirez-Campillo R., Vieira C.A., Gentil P. (2018). Can We Draw General Conclusions from Interval Training Studies?. Sports Med..

[18] Komi P.V., Linnamo V., Silventoinen P., Sillanpaa M. (2000). Force and EMG power spectrum during eccentric and concentric actions. Med. Sci. Sports Exerc..

[19] Margaritelis N.V., Theodorou A.A., Chatzinikolaou P.N., Kyparos A., Nikolaidis M.G., Paschalis V. (2020). Eccentric exercise per se does not affect muscle damage biomarkers: Early and late phase adaptations. Eur. J. Appl. Physiol..

[20] Paschalis V., Nikolaidis M.G., Theodorou A.A., Panayiotou G., Fatouros I.G., Koutedakis Y., Jamurtas A.Z. (2011). A weekly bout of eccentric exercise is sufficient to induce health-promoting effects. Med. Sci. Sports Exerc..

[21] Theodorou A.A., Panayiotou G., Paschalis V., Nikolaidis M.G., Kyparos A., Mademli L., Grivas G.V., Vrabas I.S. (2013). Stair descending exercise increases muscle strength in elderly males with chronic heart failure. BMC Res. Notes.

[22] Franchi M.V., Reeves N.D., Narici M.V. (2017). Skeletal Muscle Remodeling in Response to Eccentric vs. Concentric Loading: Morphological, Molecular, and Metabolic Adaptations. Front. Physiol..

[23] Groeber M., Reinhart L., Kornfeind P., Baca A. (2019). The Contraction Modalities in a Stretch-Shortening Cycle in Animals and Single Joint Movements in Humans: A Systematic Review. J. Sports Sci. Med..

[24] Abbott B.C., Bigland B., Ritchie J.M. (1952). The physiological cost of negative work. J. Physiol..

[25] Beltman J.G., Sargeant A.J., van Mechelen W., de Haan A. (2004). Voluntary activation level and muscle fiber recruitment of human quadriceps during lengthening contractions. J. Appl. Physiol..

[26] Bigland-Ritchie B., Woods J.J. (1976). Integrated electromyogram and oxygen uptake during positive and negative work. J. Physiol..

[27] Dufour S.P., Lampert E., Doutreleau S., Lonsdorfer-Wolf E., Billat V.L., Piquard F., Richard R. (2004). Eccentric cycle exercise: Training application of specific circulatory adjustments. Med. Sci. Sports Exerc..

[28] Joumaa V., Herzog W. (2013). Energy cost of force production is reduced after active stretch in skinned muscle fibres. J. Biomech..

[29] Lombardi V., Piazzesi G. (1990). The contractile response during steady lengthening of stimulated frog muscle fibres. J. Physiol..

[30] Penailillo L., Blazevich A., Numazawa H., Nosaka K. (2013). Metabolic and muscle damage profiles of concentric versus repeated eccentric cycling. Med. Sci. Sports Exerc..

[31] Perrey S., Betik A., Candau R., Rouillon J.D., Hughson R.L. (2001). Comparison of oxygen uptake kinetics during concentric and eccentric cycle exercise. J. Appl. Physiol..

[32] Ryschon T.W., Fowler M.D., Wysong R.E., Anthony A., Balaban R.S. (1997). Efficiency of human skeletal muscle in vivo: Comparison of isometric, concentric, and eccentric muscle action. J. Appl. Physiol..

[33] Fortuna R., Groeber M., Seiberl W., Power G.A., Herzog W. (2017). Shortening-induced force depression is modulated in a time- and speed-dependent manner following a stretch-shortening cycle. Physiol. Rep..

[34] Fukutati A., Misaki J., Isaka T. (2017). Both the elongation of attached crossbridges and residual force enhancement contribute to joint torque enhancement by the stretch-shortening cycle. R. Soc. Open Sci..

[35] Herzog W. (2018). The multiple roles of titin in muscle contraction and force production. Biophys. Rev..

[36] Lindstedt S.L., La Stayo P.C., Reich T.E. (2001). When active muscles lengthen: Properties and consequences of eccentric contractions. News Physiol. Sci..

[37] Barstow T.J. (2019). Understanding near infrared spectroscopy and its application to skeletal muscle research. J. Appl. Physiol..

[38] Ferrari M., Mottola L., Quaresima V. (2004). Principles, techniques, and limitations of near infrared spectroscopy. Can. J. Appl. Physiol..

[39] Buchheit M., Cormie P., Abbiss C.R., Ahmaidi S., Nosaka K.K., Laursen P.B. (2009). Muscle deoxygenation during repeated sprint running: Effect of active vs. passive recovery. Int. J. Sports Med..

[40] Kriel Y., Kerherve H.A., Askew C.D., Solomon C. (2016). The Effect of Active versus Passive Recovery Periods during High Intensity Intermittent Exercise on Local Tissue Oxygenation in 18–30 Year Old Sedentary Men. PLoS One.

[41] Racinais S., Bishop D., Denis R., Lattier G., Mendez-Villaneuva A., Perrey S. (2007). Muscle deoxygenation and neural drive to the muscle during repeated sprint cycling. Med. Sci. Sports Exerc..

[42] Smith K.J., Billaut F. (2012). Tissue oxygenation in men and women during repeated-sprint exercise. Int. J. Sports Physiol. Perform..

[43] Woorons X., Dupuy O., Mucci P., Millet G.P., Pichon A. (2019). Cerebral and Muscle Oxygenation during Repeated Shuttle Run Sprints with Hypoventilation. Int. J. Sports Med..

[44] Zafeiridis A., Kounoupis A., Dipla K., Kyparos A., Nikolaidis M.G., Smilios I., Vrabas I.S. (2015). Oxygen Delivery and Muscle Deoxygenation during Continuous, Long- and Short-Interval Exercise. Int. J. Sports Med..

[45] Denis R., Bringard A., Perrey S. (2011). Vastus lateralis oxygenation dynamics during maximal fatiguing concentric and eccentric isokinetic muscle actions. J. Electromyogr. Kinesiol..

[46] Muthalib M., Lee H., Millet G.Y., Ferrari M., Nosaka K. (2010). Comparison between maximal lengthening and shortening contractions for biceps brachii muscle oxygenation and hemodynamics. J. Appl. Physiol..

[47] Okamoto T., Masuharab M., Ikutac K. (2006). Differences of muscle oxygenation during eccentric and concentric contraction. Iso. Exerc. Sci..

[48] Secher N.H., Seifert T., Van Lieshout J.J. (2008). Cerebral blood flow and metabolism during exercise: Implications for fatigue. J. Appl. Physiol..

[49] Subudhi A.W., Dimmen A.C., Roach R.C. (2007). Effects of acute hypoxia on cerebral and muscle oxygenation during incremental exercise. J. Appl. Physiol..

[50] Rupp T., Perrey S. (2008). Prefrontal cortex oxygenation and neuromuscular responses to exhaustive exercise. Eur. J. Appl. Physiol..

[51] Shibuya K., Kuboyama N. (2010). Decreased activation in the primary motor cortex area during middle-intensity hand grip exercise to exhaustion in athlete and nonathlete participants. Percept. Mot. Ski..

[52] Shibuya K., Tanaka J., Kuboyama N., Ogaki T. (2004). Cerebral oxygenation during intermittent supramaximal exercise. Respir. Physiol. Neurobiol..

[53] Beam W.C., Adams G.M. (2002). Exercise Physiology Laboratory Manual.

[54] Dolopikou C.F., Kourtzidis I.A., Margaritelis N.V., Vrabas I.S., Koidou I., Kyparos A., Theodorou A.A., Paschalis V., Nikolaidis M.G. (2020). Acute nicotinamide riboside supplementation improves redox homeostasis and exercise performance in old individuals: A double-blind cross-over study. Eur. J. Nutr..

[55] Grassi B., Quaresima V. (2016). Near-infrared spectroscopy and skeletal muscle oxidative function in vivo in health and disease: A review from an exercise physiology perspective. J. Biomed. Opt..

[56] Matcher S.J., Elwell C.E., Cooper C.E., Cope M., Delpy D.T. (1995). Performance comparison of several published tissue near-infrared spectroscopy algorithms. Anal. Biochem..

[57] Cardinale M., Ferrari M., Quaresima V. (2007). Gastrocnemius medialis and vastus lateralis oxygenation during whole-body vibration exercise. Med. Sci. Sports Exerc..

[58] van Beekvelt M.C., Borghuis M.S., van Engelen B.G., Wevers R.A., Colier W.N. (2001). Adipose tissue thickness affects in vivo quantitative near-IR spectroscopy in human skeletal muscle. Clin. Sci..

[59] Borg G.A. (1973). Perceived exertion: A note on "history" and methods. Med. Sci. Sports.

[60] Buchheit M., Abbiss C.R., Peiffer J.J., Laursen P.B. (2012). Performance and physiological responses during a sprint interval training session: Relationships with muscle oxygenation and pulmonary oxygen uptake kinetics. Eur. J. Appl. Physiol..

[61] Burgomaster K.A., Heigenhauser G.J., Gibala M.J. (2006). Effect of short-term sprint interval training on human skeletal muscle carbohydrate metabolism during exercise and time-trial performance. J. Appl. Physiol..

[62] Gonzalez-Izal M., Lusa Cadore E., Izquierdo M. (2014). Muscle conduction velocity, surface electromyography variables, and echo intensity during concentric and eccentric fatigue. Muscle Nerve.

[63] Paulus J., Croisier J.L., Kaux J.F., Bury T. (2019). Eccentric versus Concentric - Which Is the Most Stressful Cardiovascularly and Metabolically?. Curr. Sports Med. Rep..

[64] Billaut F., Davis J.M., Smith K.J., Marino F.E., Noakes T.D. (2010). Cerebral oxygenation decreases but does not impair performance during self-paced, strenuous exercise. Acta Physiol..

[65] Monroe D.C., Gist N.H., Freese E.C., O’Connor P.J., McCully K.K., Dishman R.K. (2016). Effects of Sprint Interval Cycling on Fatigue, Energy, and Cerebral Oxygenation. Med. Sci. Sports Exerc..

[66] Santos-Concejero J., Billaut F., Grobler L., Olivan J., Noakes T.D., Tucker R. (2017). Brain oxygenation declines in elite Kenyan runners during a maximal interval training session. Eur. J. Appl. Physiol..

[67] Puhan M.A., Büsching G., Schünemann H.J., VanOort E., Zaugg C., Frey M. (2006). Interval versus continuous high-intensity exercise in chronic obstructive pulmonary disease: A randomized trial. Ann. Intern. Med..

[68] Davies R.C., Eston R.G., Poole D.C., Rowlands A.V., DiMenna F., Wilkerson D.P., Twist C., Jones A.M. (2008). Effect of eccentric exercise-induced muscle damage on the dynamics of muscle oxygenation and pulmonary oxygen uptake. J. Appl. Physiol..

